# Effect of Layer Charge Characteristics on the Distribution Characteristics of H_2_O and Ca^2+^ in Ca-Montmorillonites Interlayer Space: Molecular Dynamics Simulation

**DOI:** 10.3390/ma12142318

**Published:** 2019-07-20

**Authors:** Jun Qiu, Guoqing Li, Dongliang Liu, Shan Jiang, Guifang Wang, Ping Chen, Xiangnan Zhu, Xiaoqiang Cao, Xianjun Lyu

**Affiliations:** 1 College of Chemical and Environmental Engineering, Shandong University of Science and Technology, Qingdao 266590, China; 2 School of Resources Environment and Materials, Guangxi University, Nanning 530004, China

**Keywords:** montmorillonite, charge densities, charge position, distribution characteristics, molecular dynamics simulation

## Abstract

The charge characteristics of montmorillonite have significant effects on its hydration and application performances. In this study, a molecular dynamics simulation method was used to study the influence of the charge position and charge density of montmorillonite on the distribution of H_2_O and Ca^2+^ in layers. The results showed that when the layer charge is mainly derived from the substitution among ions in the tetrahedron, a large number of H_w_ and O_t_ are combined into a hydrogen bond in the interlayer, thus the water molecules are more compactly arranged and the diffusion of water molecules among the layers is reduced. In addition, the Ca^2+^ are diffused to the sides by a concentrated distribution in the central axis of the layer. As the charge density of the montmorillonite increases, the polarity of the Si–O surface increases, which lesds to the deterioration of the diffusibility of the water molecules and the structure of the water molecules in the interlayers is more stable. The increase in the layer charge density lesds to the expansion of the isomorphic substitution range of the crystal structure, which results in a more dispersed distribution of Ca^2+^ among the layers under the action of electrostatic attraction between the substituted negative sites and the Ca^2+^.

## 1. Introduction

Montmorillonite is a typical clay mineral with a unique crystal structure, which provides it with a high cation exchange capacity and high expansion capacity, thus, it is widely used in various fields. The molecular simulation method used in this study is useful and has several advantages [1,2,3]. Not only can it simulate the molecular structure of the material [4], it can also simulate the dynamic change of molecules [5,6], intuitively describe the mechanism of the chemical reaction at the molecular and atomic scale, and verify the rationality of conclusions or predict the results of experiments [7,8]. Therefore, many scholars have also used the molecular simulation method to study the hydration properties of montmorillonite [9].

The research study on montmorillonite using molecular simulation technology began in the 1990s. Skipper et al. first used Monte Carlo (MC) and molecular dynamics (MD) methods to simulate the interlayer water of Mg-based montmorillonite (Mg-MMT), and obtained the interlayer spacing of Mg-MMT under different water content conditions [10,11]. When the simulation reached equilibrium, the layer spacing of Mg-MMT was 14.7 ± 0.1 Å, which was very close to the actual measured value of 15.1 Å by XRD (X-Ray Diffraction analysis) [12]. After that, Boek et al. successfully explained the water adsorption process of montmorillonite and the expansion of montmorillonite by means of computer simulation [11,13]. In view of the phenomenon that the interlayer height of Na-montmorillonite (Na-MMT) increased with the increasing of water content, the interlayer spacing of Na-MMT with different water content was simulated, and the simulation results were very consistent with the XRD data. Mary et al. used the Monte Carlo method and the molecular dynamics method to study the Na-MMT and Cs-MMT, and compared the structure and dynamics characteristics with the experimental data, the results showed that the calculated layer distance and the diffusion coefficient of water molecules in single hydrate and interlayer cations were basically identical by the experimental data [14]. Therefore, as a new research method, molecular dynamics simulation has been widely used in the study of montmorillonite [15].

Nowadays, many scholars have studied the hydration properties of montmorillonite using molecular simulation technology and have achieved some gratifying results in this field. Seppälä et al. studied the influence of layer charge on the structure of interlayer water and diffusion of water and cations by the methods of DFT (Density Functional Theory) and molecular dynamics, and found that the diffusion coefficient increased with the increase of water content and decrease of layer charge [16]. Greathouse studied the diffusion mechanism of water and cations in montmorillonite interlayers. The result showed that calculated activation energies for ion and water diffusion in Na-montmorillonite were similar to each other and to the water hydrogen bond energy, suggesting the breaking of water–water and water-clay hydrogen bonds as a likely mechanism for interlayer diffusion [3]. Miranda-Pascuala used the Monte Carlo method to intensively study the expansion characteristics of Na-MMT, Mg-MMT, and Na/Mg-MMT, and found that when there were multiple cations existing in the interlayer of montmorillonite, the properties of montmorillonite mainly depended on the cations with the highest content in the interlayer of montmorillonite [17]. Zhang et al. used molecular dynamics simulations to investigate the swelling properties, hydration behaviors, and mobility of the interlayer species of (Nax, Cay)-montmorillonites with different water contents. The results showed that in all montmorillonites, the mobility of Na^+^ was always much greater than that of Ca^2+^ due to their different hydration shells, Ca^2+^ hydration complexes were pronounced more stable than those of Na^+^, and in montmorillonites with high Ca^2+^/Na^+^ ratio, the inhibitory effects of Ca^2+^ hydration complexes on the mobility of Na^+^ were clearly revealed [18].

In the mechanism of the influence of ions on the hydration characteristics in montmorillonite interlayers, most researchers believe that montmorillonite has high swelling capacity and hydrophilicity when montmorillonite layers contain cations with strong hydration ability (such as Na^+^ and Li^+^) [3,17,19,20]. Mary and Mignon used the Monte Carlo method and the molecular dynamics method to study the behavioral characteristics of different cations (Li^+^, Na^+^, and K^+^) in the process of montmorillonite hydration, and found that with the increasing of the montmorillonite interlayer water content, Na^+^ and Li^+^ were easy to separate from the interlayer of montmorillonite, while K^+^ would move and be bound to the silicon oxygen tetrahedron surface [21,22]. On the contrary, when the layers contained cations with weak hydration ability (such as K^+^ and Cs^+^), montmorillonite had weak expansion ability and poor hydrophilicity, and easily was affected by various factors such as temperature and pressure [23,24,25].

The charge characteristics of montmorillonite with different origins are quite different [20,26,27,28,29], which is not only reflected in the charge densities, but also in the substitution amount of tetrahedral central ions and octahedral central ions in the montmorillonite crystal structure. At present, there are few studies about the interlayer water molecules and calcium ions distribution characteristics of Ca-montmorillonite and their influencing mechanism by molecular dynamics simulation. In this study, according to the layer charge density distribution characteristics of Ca-montmorillonite in nature, we used molecular simulation to establish the montmorillonite crystal structure models with different layer charge characteristics, and used molecular dynamics as a powerful computational tool to study the influence mechanism of montmorillonite layer charge density and inter-ion substitution position on the distribution characteristics of water molecules and Ca^2+^ in the interlayer.

## 2. Simulation Details

### 2.1. Simulation Model Establishment

In this study, Materials Studio 7.0 simulation software was used to simulate the distribution characteristics of Ca^2+^ and water molecules in Ca-montmorillonite with different charge density [30]. Material Studio is a material simulation computing platform developed by Accelrys, USA. Firstly, according to the semi-crystal structure formula M_x+y_(Al_2–x_Mg_x_)(Si_4–y_Al_y_)O_10_(OH)_2_nH_2_O, the corresponding montmorillonite crystal model was established [31]. Where M is the interlayer exchangeable cation (Ca^2+^) and x + y is the unit layer charge. The structure belongs to the monoclinic C2/m space group, the crystal layer constant a = 0.523 nm, b = 0.906 nm, and the c value is variable, when the structural unit layer is anhydrous, c = 0.960 nm [32]; if water molecules are present among the structural unit layers, the value of c will vary with the number of water molecules and the exchangeable cations in the interlayers. Table 1 shows the atomic coordinates of montmorillonite. Secondly, based on these previously mentioned parameters, the montmorillonite model can be established. The montmorillonite super-cell consisted of 32 formula units in the form of 8 × 4 × 1 in the X-,Y- and Z-axis directions, respectively. Thirdly, according to our studying purpose, we designed different isomorphic substitution conditions in the super-cell. The principle of substitution is: the substitution position is random and two adjacent positions can’t be replaced at the same time.

According to the crystal structure characteristics of natural montmorillonite, combined with the research purpose of this paper, the layer charge density of the Ca-montmorillonite semi-cell is 0.25, 0.375, 0.5, 0.625, respectively, and the following were recorded as M1, M2, M3, and M4. The substitution positions occurred in octahedron and tetrahedron, respectively. The ratio of the number of octahedral substitution sites to tetrahedral substitution sites is: 0:6, 2:4, 3:3, 4:2, 6:0, the following are recorded as 0-6, 2-4, 3-3, 4-2, 6-0, eventually twenty montmorillonite crystal models were established. For example, for a montmorillonite with a layer charge density of 0.25, the ratio of the octahedral to tetrahedral substitution sites is 0:6, which is recorded as M1_0-6_. 

**Figure 1:**
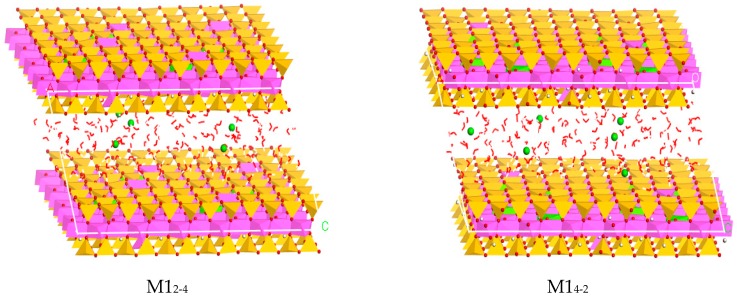
The different crystal structure models of montmorillonites with different layer charge positions (using M1_2-4_ and M1_4-2_ as two examples, the green color in octahedron represents the substitution position, the pink color in tetrahedron represents the substitution position).

The water molecules among the TOT (Tetrahedron-Octahedron-Tetrahedron) crystal layers are added by the Adsorption Location module. Since the amount of water molecules in the interlayers can affect the interlayer spacing of the montmorillonite, when the montmorillonite contains a layer of water molecules, the layer spacing is about 12.5 Å, and the two layers are about 15.0 Å [33,34]; in order to study the influence of layer charge characteristics of montmorillonite on water molecules and Ca^2+^ in the interlayer, this paper used the established 8a × 4b × 1c super-cell to simulate. Two-hundred-and-fifty-six water molecules were added into the montmorillonite model to form two layers of water molecules, and the corresponding Ca–montmorillonite layer spacing was 15.0 Å [35]. Corresponding to four types of Ca–montmorillonites with different charge densities, five different ion substitution modes and substitution ratios, twenty montmorillonite hydration models were finally established (taking M1 as an example, as shown in Figure 1).

### 2.2. Simulation Parameters

In the geometric optimization, it was assumed that the TOT sheets of the calcium-based montmorillonite remained rigid, the unit cell parameters a, b, α, and γ remained unchanged, and c and β were variables [36,37]. In this study, the Smart Minimizer algorithm was selected. The parameters were set to the following: the RMS Force was 0.1 kcal/mol·Å, the Entergy Difference was 2 × 10^−5^ kcal/mol, the RMS Displacement was 1 × 10^−5^ Å, and the RMS Stress was 100 kPa. The Ewald summation method was for long-range electrostatic interaction and the atom-based summation method as for the short-range van der Waals forces. The vacuum cutoff spacing was 12.5 Å, the spline width was 1.0 Å, the buffer width was 0.5 Å, and the number of iteration steps was 5000.

The molecular dynamics simulation used the Forcite module and the NPT and NVT ensembles. The temperature was 298.0 K, the step length was 0.5 fs, the total simulation time of the NPT was 100 ps, and the total simulation time of the NVT was 150 ps. The first 50 ps was used for simulation and the last 100 ps was used for analysis. The number of steps was 1 × 10^5^ and the output was every 200 steps. The universal force field (UFF) was used for simulation in this work [38]. The UFF is the force field that basically covers all the elements in the periodic table of elements, which can meet the technical requirements for the simulation in this paper. During the simulation, all atoms and ions in the interlayer were unconstrained, allowing atomic coordinates and lattice parameters to change freely, and the output data were used for result analysis. The basic parameters of the twenty Ca-montmorillonite models are shown in the following Table 2.

## 3. Analysis and Discussion

The distribution characteristics of water molecules and calcium ions in the montmorillonite layer during molecular dynamics simulation can be visually and quantitatively described by the Z-axis density distribution curves, mean square displacement function (MSD), and radial distribution function (RDF) of the final configuration.

### 3.1. Z-Axis Density Distribution

By molecular dynamics simulations, the Z-axis density distribution curves reflects the density distribution of the components in the montmorillonite interlayer in the direction of the vertical montmorillonite layer. Thus, the distribution and relative position of water molecules and Ca^2+^ in the interlayer of montmorillonite can be further studied, and the influence mechanism of charge density of montmorillonite on interlayer particles can be explained.

#### 3.1.1. Z-Axis Density Distribution Characteristics of Water Molecules

**Figure 2:**
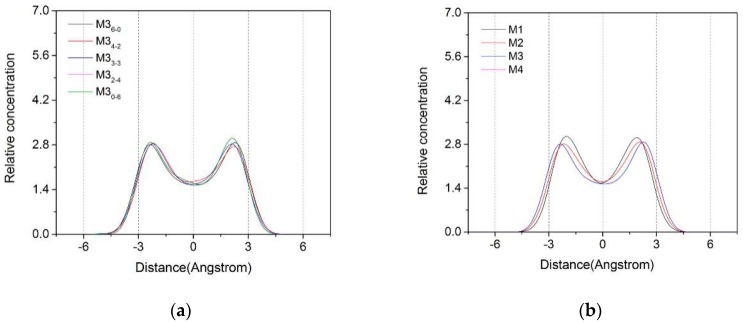
The Z-axis density distribution of water molecules in the interlayers with different layer charge characteristics, (**a**) charge position, M3; (**b**) charge density, Mi_0–6_.

The Z-axis density distribution curves of water molecules in montmorillonite layers with different layer charge characteristics are shown in Figure 2. It can be seen from the left figure that the Z-axis density distribution of water molecules in the interlayer of Ca-montmorillonite has little difference, and the overall distribution was bimodal. The two peaks were distributed in the range of ±2.5 Å on both sides of the central axis among the two TOT sheets and were substantially symmetrical. Therefore, the different substitution sites had no significant effect on the Z-axis density distribution of water molecules. Figure 2b shows that the layer charge substitution sites had the same characteristics, as the charge density increased from 0.25 to 0.625, The water molecules in the montmorillonite layer move along the axis of the center. Moreover, the water molecules’ distribution range extended to both sides along the central axis, the left side moved from −1.7 Å to −2.5 Å, and the right side moved from 1.8 Å to 2.5 Å. The reason was that as the charge density of the montmorillonite layer increased, the polarity of the siloxane surface was strengthened, and the water molecules moved toward the siloxane surface on both sides of the interlayer by the hydrogen bonding force between the oxygen in the siloxane surface and the hydrogen in the water molecules.

#### 3.1.2. Z-Axis Density Distribution Characteristics of Ca^2+^

**Figure 3:**
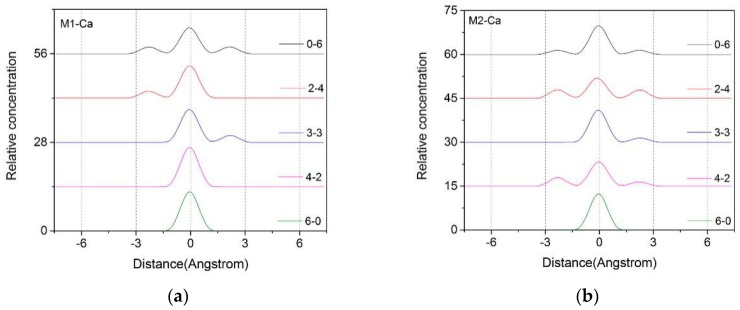

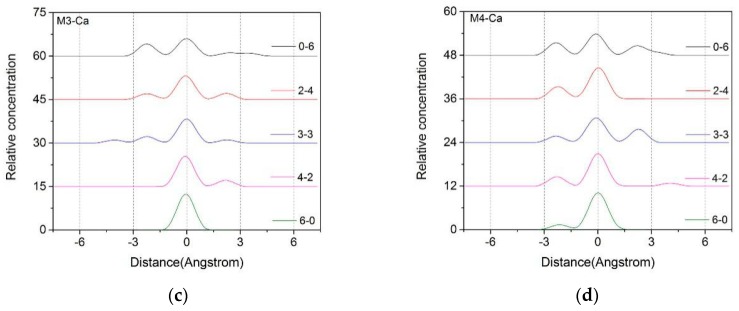
The Z-axis density distribution of Ca^2+^ in the interlayers of montmorillonite with different layer charge characteristics. (**a**) M1; (**b**) M2; (**c**) M3; (**d**) M4.

The Z-axis density distribution curves of Ca^2+^ in the montmorillonite layers with different layer charge characteristics are shown in Figure 3. From the figure we can find that the Ca^2+^ in the interlayer were mainly concentrated in the range of −1.5 Å ~ +1.5 Å along the central axis of the montmorillonite. The change in the charge position in montmorillonite had a certain influence on the distribution of Ca^2+^ in the interlayer. When the layer charge as derived from aluminum-substituted silicon in the octahedron, such as Mi_6-0_, the Ca^2+^ in the layers mainly showed a single peak and were concentrated in the range of −1.5 Å ~ +1.5 Å in the interlayer domain. In addition, the different charge densities of the montmorillonite had little difference in the Z-axis density distribution of Ca^2+^, indicating that the layer charge negative potential in the octahedron had little effect on the distribution characteristics of interlayer Ca^2+^ because of the long-range spatial effect. As the amount of Mg^2+^ substituted Al^3+^ in the tetrahedron increases, the amount of Al^3+^ substituted Si^4+^ in the corresponding octahedron decreases, the Ca^2+^ will shift toward the siloxane surface on both sides in the interlayers, and the Z-axis density distribution of Ca^2+^ will show a bimodal or trimodal distribution. The phenomenon was more pronounced in the high-charged montmorillonite such as M2, M3, and M4. Due to the short-term effect of space, the Ca^2+^ was closer to the negative charge position in the tetrahedron in the interlayer, as part of the Ca^2+^ were subjected to a large electrostatic attraction and moved toward the tetrahedron, so the Z-axis distribution of Ca^2+^ exhibited a multi-peak distribution.

In addition, the charge density of montmorillonite had a significant effect on the distribution of Ca^2+^ in the interlayer from Figure 3. With the increase of the layer charge density, the Ca^2+^ moved more significantly from the middle of the layer to the siloxane surface on both sides, as shown in the figure, migration peaks increased or multiple migration peaks appeared. This phenomenon was more pronounced as the proportion of the charge in the tetrahedron increased. The Ca^2+^ among the layers were subjected to the negative potential electrostatic attraction of the isomorphism of the tetrahedron, causing the Ca^2+^ to diffuse to the sides. Since the montmorillonite with higher charge density had a large number of negative potential points, the Ca^2+^ in the interlayer were subjected to strong electrostatic force, and the diffusion to the siloxane surface was more obvious. Therefore, in the same crystal structure of montmorillonite, with the increase of the charge density of montmorillonite, the Ca^2+^ appeared to gradually shift from the vicinity of the central axis to the two sides in the montmorillonite layer.

### 3.2. Mean Square Displacement (MSD) and Self-Diffusion Coefficient

The mean square displacement is the mean square of the change in particle position in respect to its initial position at different times, which can reflect the change of particle offset with time [39]. The diffusion coefficient reflects the mobility of the particles, indicating the magnitude of the change in particle position with time, and its magnitude can be derived from the mean square displacement [40]. By analyzing the diffusion of water molecules and Ca^2+^ between Ca-montmorillonite layers, the microscopic mechanism of macroscopic hydration characteristics exhibited by montmorillonite can be studied, which provides theoretical guidance for the deep processing and application of montmorillonite. The self-diffusion coefficient of interlayer particles in the montmorillonite interlayer can be derived from the mean square displacement curve of the particle, and the magnitude of the self-diffusion coefficient is proportional to the slope of the mean square displacement curve, i.e., [41]:

(1) $$D=\frac{1}{6N_{\alpha }}\underset{t\to \infty }{\lim}\frac{d}{dt}{\left|r_{i}\left(t\right)-r_{j}\left(0\right)\right|}^{2}$$

where ${\left|r_{i}\left(t\right)-r_{j}\left(0\right)\right|}^{2}$ is the mean square displacement of the molecule, *N_α_* is the total number of α particles, *r_i_* denotes the position vector of *i*th particle and the angular brackets denote an ensemble average, and the self-diffusion coefficient D is 1/6 of the slope of the mean square displacement curve.

#### 3.2.1. MSD and Self-Diffusion Coefficient of Water Molecules

**Figure 4:**
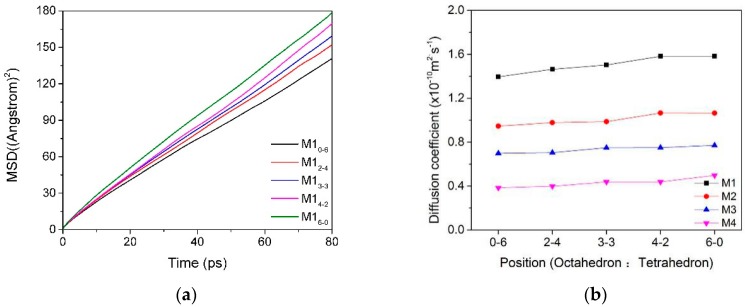
The MSD ((**a**), M1) and self-diffusion coefficient (**b**) of water molecules in the interlayer of montmorillonite with different charge characteristics.

The mean square displacement curves and self-diffusion coefficients of water molecules in the interlayer of the Ca-montmorillonite with different charge characteristics are shown in Table 3 and Figure 4. We can find that the charge position and density of montmorillonite have a certain influence on the diffusibility of water molecules in the interlayer. With the proportion of charge density produced by montmorillonite octahedral substitution increased, the mobility of water molecules in the inter-layer domain was higher and, thus, had a higher diffusion coefficient (Figure 4a, M1). It can be inferred that the self-diffusion coefficient of water molecules was affected by the polarity of the montmorillonite siloxane surface. In other words, if the total charge was fixed, when the charge source was generated in the tetrahedron, due to the short-range spatial effect, the water molecules were subjected to strong polar attraction and difficult to move, so the self-diffusion coefficient was low.

Compared with the influence of the charge position, the charge density of the montmorillonite had a more significant effect on the diffusibility of the water molecules in the interlayers, and the diffusibility of the water molecules decreased with the increase in the layer charge density (Figure 4b). The reason was that as the charge density of montmorillonite increased, the polar attraction of siloxane to water molecules increased; water molecules moved toward the siloxane surface, and the number of hydrogen bonds formed by the combination of O in the siloxane surface (O_t_) and H in the water molecule (H_w_) increased. This is consistent with Greathouse’s conclusion that water molecular diffusivity is related to hydrogen bond fracture among layers of montmorillonite [3]. Due to the action of hydrogen bonding, water molecules are bound to the surface of the montmorillonite tetrahedron and difficult to diffuse. This phenomenon was more pronounced when the ratio of tetrahedral isomorphism was higher in montmorillonite.

#### 3.2.2. MSD and Self-Diffusion Coefficient of Ca^2+^

**Figure 5:**
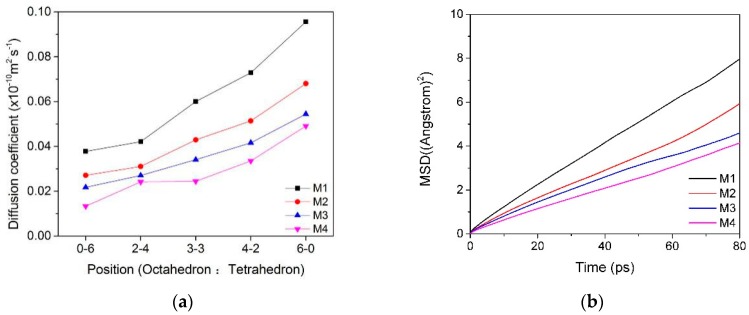
The MSD ((**a**), Mi_0-6_) and self-diffusion coefficient ((**b**), M_i_) of Ca^2+^ in the interlayer of montmorillonite with different charge characteristics.

The mean square displacement curves and self-diffusion coefficients of Ca^2+^ in the interlayer of the Ca–montmorillonite with different charge characteristics are shown in Table 4 and Figure 5. We can see that the self-diffusion coefficients of Ca^2+^ were obviously affected by the position of the lattice substitution (Figure 5a). When the layer charge of montmorillonite was mostly formed in a tetrahedron, the diffusion coefficient of Ca^2+^ was lower than that formed in an octahedron because of the short-range effect, as mentioned in Section 3.1.2. The influence of charge density on the diffusion of Ca^2+^ is shown in Figure 5b; as the charge density of montmorillonite increased, the electrostatic attraction between the Ca^2+^ and the Si–O surface strengthened, as a result, the diffusion coefficient of Ca^2+^ was lower. This is consistent with Seppälä’s [16] conclusion that the diffusion coefficient of calcium ions decreases with the increase in the layer charge.

In summary, Table 3 and Table 4 show that the diffusivity of water molecules among montmorillonite layers was much larger than that of Ca^2+^, but the diffusion coefficient of Ca^2+^ was more affected by the charge position (Figure 4 and Figure 5), which was because the water molecules were neutral, although the polar side (the side where H was located) would be affected by the negative side of the siloxane surface. However, compared to the Ca^2+^ with positive charge, the polarity of the water molecules was much weaker. Thus, water molecules in the interlayer diffused more easily than Ca^2+^. As for the influence from an overall point of view, when the charge density of montmorillonite was high or the ratio of tetrahedral substitution position was high, the hydration performance of montmorillonite was worse due to the low diffusibility of water molecules and Ca^2+^.

### 3.3. The Radial Distribution Function (RDF)

The radial distribution function reflects the probability density among two types of atoms and can reflect the aggregation characteristics of ions in the system [42]. By analyzing the radial distribution function among two different particles in the montmorillonite interlayer domain, we can infer whether there is a force among the two types of atoms and the magnitude of their force. The radial distribution function of the system is the algebraic averaging of the radial distribution functions of all the same atoms, reflecting the atomic distribution characteristics of the system [39]. The calculation formula is:

(2) $$g_{\alpha \beta }\left(r\right)=n_{\beta }/4\pi \rho_{\beta }r^{2}dr$$

where *g_αβ_*(*r*) is the radial distribution of *β*; *n_β_* is the number of β with a radius of *r* → *r* + *dr*; *ρ_β_* is the number density of *β*; and *r* is the distance between *α* and *β*. In this work, the characteristics of the radial distribution function of oxygen in the siloxane surface (O_t_) and hydrogen atoms in the water molecules (H_w_) and Ca^2+^ in the interlayer domain were studied.

#### 3.3.1. The RDF of O_t_-H_w_

**Figure 6:**
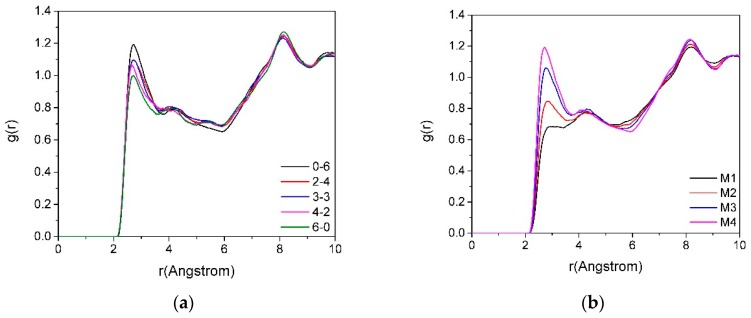
The RDFs of O_t_-H_w_ in montmorillonite with different charge positions ((**a**), M4) and densities ((**b**), Mi_0-6_).

Figure 6 provides the RDFs of O_t_-H_w_ in montmorillonite with different charge positions (Figure 6a, M4) and densities (Figure 6b, Mi_0-6_). The g (O_t_-H_w_) presents the RDF of O_t_ and H_w_. Figure 6a shows that first peak of g(O_t_-H_w_) of montmorillonite appears at the distance of approximately 2.5 Å. With the increase of the ratio of isomorphism in the tetrahedron, the intensity of the main peak of g(O_t_-H_w_) was enhanced, which indicates that when the montmorillonite layer charge was mostly derived from the substitution among ions in the tetrahedron, the Si–O surface exhibited a stronger polar force, and the water molecule had a polar side (i.e., the side where H was located), which was subjected to a stronger polar force from the Si–O surface, making the water molecules more compact near the siloxane surface, so the intensity of the main peak of g(O_t_-H_w_) in the RDF was enhanced.

In Figure 6b, the g(O_t_-H_w_) shows the RDF between O_t_ and H_w_ in the interlayer with different charge densities, which indicates that as the layer charge density increased, the intensity of the first main peak of g(O_t_-H_w_) gradually increased. The reason was that the polarity of the Si–O surface increases with the increase in the charge density of the montmorillonite, and the H_w_ was pulled by a stronger polar force when the layer charge was higher; therefore, the first main peak of g(O_t_-H_w_) gradually increased with an increase in the layer charge density. This further demonstrates that the diffusivity of water molecules among the layers of montmorillonite decreased as the layer charge density increased.

#### 3.3.2. The RDF of O_t_-Ca

**Figure 7:**
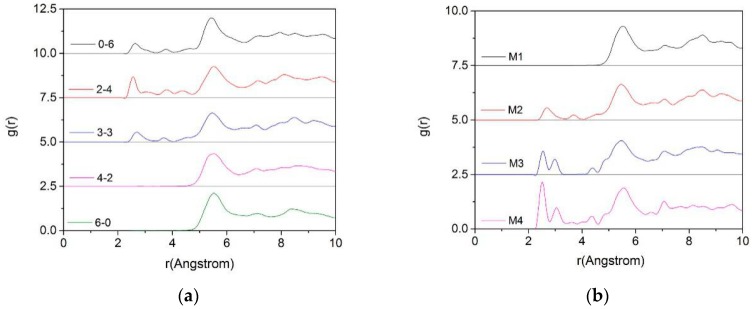
The RDF of O_t_-Ca of montmorillonite with different charge positions ((**a**), M2) and charge densities ((**b**), Mi_3-3_).

Figure 7 shows the RDFs of O_t_-Ca in montmorillonite with different charge positions (Figure 7a, M2) and densities (Figure 7b, Mi_3-__3_).

As can be seen from the left figure, if the layer charge density is fixed, the difference in the substitution positions will significantly affect the distribution of Ca^2+^. When ion substitution occurred, mostly in tetrahedrons (0-6, 2-4, 3-3), Ca^2+^ were shifted closer to the Z-axis due to their closer proximity to the substitution source, and the distribution was more uniform. A low peak appeared at a distance (2.8 Å) closer to the O_t_, but most of the Ca^2+^ were still concentrated in the middle of the layer, about 5.5 Å from the O_t_. When the substitution source was mostly in octahedrons (4-2, 6-0), the radial distribution of Ca^2+^ was less affected due to the spatial long-range effect mentioned in Section 3.1.2.

From Figure 7b, we can find that with the increase in the charge density of montmorillonite, due to the negative charge of the Si–O surface increases, some of the Ca^2+^ moved toward the Si–O surface under the action of electrostatic attraction. Therefore, some new peaks appearred around 2.5 Å–3.5 Å, which was more obvious when the layer charge density of the montmorillonite was higher (M3, M4).

## 4. Conclusions

By simulating the distribution characteristics of water molecules and Ca^2+^ in the interlayer under different charge characteristics of montmorillonite, the following conclusions were obtained:

(1) When the layer charge of the Ca-montmorillonite was mainly derived from the replacement of Si^4+^ with Al^3+^ in the tetrahedron, the H_w_ and the O_t_ were more easily combined to form a hydrogen bond in the interlayer. Due to the hydrogen bonding force, the water molecules were more compactly arranged, and the diffusion of water molecules among the layers was reduced. Due to the action of electrostatic attraction between the Ca^2+^ and the Si–O surface, the Ca^2+^ move to the Si–O surface on both sides and the distribution range was expanded.
(2) With the increase of the charge density in the Ca-montmorillonite layer, the polarity of the Si–O surface was enhanced, and the polar force generated by the water molecules in the interlayers was stronger, the number of hydrogen bonds between the H_w_ and the O_t_ increased, and the self-diffusion coefficient of the water molecules decreased. For the Ca^2+^, the higher the layer charge density of montmorillonite, the larger the electrostatic force of the Ca^2+^ subjected to the negative point, and the larger the distribution range of Ca^2+^.

On the basis of this work, we have a preliminary understanding of the influence of the charge-characteristic of Ca-montmorillonite on water molecules and Ca^2+^. In the future, we will conduct an in-depth study on the influence mechanism of the charge density of Ca-montmorillonite on the polarity of the Si–O surface [43]. In addition, we will further study the structure, performance, and application characteristics of organo-modified montmorillonite by means of MD. The conclusions about the influence mechanism of layer charge characteristics on the distribution characteristics of H_2_O and Ca^2+^ in Ca-montmorillonites interlayer space will give us useful information about the interlayer of montmorillonite at the atomic level.

## Figures and Tables

**Table 1:** Atomic coordinates of montmorillonite.

Atom	X	Y	Z
Al	0.000	3.020	15.500
Si	0.472	1.510	12.580
O_o_	0.122	0.000	12.040
O_o_	−0.686	2.615	12.240
O_t_	0.772	5.510	14.200
O_(OH)_	0.808	4.530	14.250
H_(OH)_	−0.103	4.530	13.812

**Table 2:** The simulation results of twenty Ca-montmorillonite models.

**M1**	**0-6**	**2-4**	**3-3**	**4-2**	**6-0**
Volume/Å^3^ × 10^3^	16.94	16.94	16.94	16.94	1.694
c/Å	15.41	15.42	15.42	15.41	15.42
β/°	104.9	105.0	105.0	105.0	105.0
n_(Hydrogen bonding)_	92	92	93	91	94
**M2**	**0-6**	**2-4**	**3-3**	**4-2**	**6-0**
Volume/Å^3^ × 10^3^	16.94	16.94	16.94	16.94	16.94
c/Å	15.41	15.42	15.42	15.42	15.41
β/°	104.9	105.0	105.0	105.0	104.9
n_(Hydrogen bonding)_	100	99	100	99	99
**M3**	**0-6**	**2-4**	**3-3**	**4-2**	**6-0**
Volume/Å^3^ × 10^3^	16.93	16.94	16.95	16.94	16.94
c/Å	15.41	15.42	15.42	15.42	15.42
β/°	105.0	105.0	105.0	104.9	105.0
n_(Hydrogen bonding)_	108	105	109	108	106
**M4**	**0-6**	**2-4**	**3-3**	**4-2**	**6-0**
Volume/Å^3^ × 10^3^	16.93	16.94	16.94	16.94	16.94
c/Å	15.42	15.41	15.42	15.42	15.42
β/°	105.1	105.0	105.0	104.9	105.0
n_(Hydrogen bonding)_	117	117	115	117	118

**Table 3:** The self-diffusion coefficient of water molecules in the interlayer of montmorillonite with different charge characteristics (×10^−10^ m^2^·s^−1^).

Type	0-6	2-4	3-3	4-2	6-0
M1	1.3959	1.4648	1.5043	1.5823	1.5823
M2	0.9461	0.9780	0.9870	1.0659	1.0650
M3	0.6987	0.7048	0.7496	0.7512	0.7717
M4	0.3848	0.3994	0.4388	0.4386	0.4977

**Table 4:** The self-diffusion coefficient of Ca^2+^ in the interlayer of montmorillonite with different charge characteristics (×10^−10^ m^2^·s^−1^).

Type	0-6	2-4	3-3	4-2	6-0
M1	0.0378	0.0421	0.0600	0.0729	0.0956
M2	0.0331	0.0311	0.0430	0.0514	0.0681
M3	0.0217	0.0270	0.0341	0.0347	0.0545
M4	0.0133	0.0242	0.0244	0.0336	0.0492

## References

[1] Mignon P., Ugliengo P., Sodupe M., Hernandez E.R. (2010). Ab initio molecular dynamics study of the hydration of Li(+), Na(+) and K(+) in a montmorillonite model. Influence of isomorphic substitution. Phys. Chem. Chem. Phys..

[2] Salles F., Douillard J.-M., Bildstein O., El Ghazi S., Prélot B., Zajac J., Van Damme H. (2015). Diffusion of Interlayer Cations in Swelling Clays as a Function of Water Content: Case of Montmorillonites Saturated with Alkali Cations. J. Phys. Chem. C.

[3] Greathouse J.A., Cygan R.T., Fredrich J.T., Jerauld G.R. (2016). Molecular Dynamics Simulation of Diffusion and Electrical Conductivity in Montmorillonite Interlayers. J. Phys. Chem. C.

[4] Park J.H., Shin H.J., Kim M.H., Kim J.S., Kang N., Lee J.Y., Kim K.T., Lee J.I., Kim D.D. (2016). Application of montmorillonite in bentonite as a pharmaceutical excipient in drug delivery systems. J. Pharm. Investig..

[5] Weng Z., Wang J., Senthil T., Wu L. (2016). Mechanical and thermal properties of ABS/montmorillonite nanocomposites for fused deposition modeling 3D printing. Mater. Des..

[6] Cygan R.T., Liang J.-J., Kalinichev A.G. (2004). Molecular Models of Hydroxide, Oxyhydroxide, and Clay Phases and the Development of a General Force Field. J. Phys. Chem. B.

[7] Shahriyari R., Khosravi A., Ahmadzadeh A. (2013). Nanoscale simulation of Na-Montmorillonite hydrate under basin conditions, application of CLAYFF force field in parallel GCMC. Mol. Phys..

[8] Tangaraj V., Janot J.M., Jaber M., Bechelany M., Balme S. (2017). Adsorption and photophysical properties of fluorescent dyes over montmorillonite and saponite modified by surfactant. Chemosphere.

[9] Anirudhan T.S., Ramachandran M. (2014). Removal of 2,4,6-trichlorophenol from water and petroleum refinery industry effluents by surfactant-modified bentonite. J. Water Process Eng..

[10] Skipper N.T. (1995). Monte carlo simulation of interlayer molecular structure in swelling clay minerals. 2. Monolayer hydrates. Clays Clay Miner..

[11] Boek E.S., Coveney P.V., Skipper N.T. (1995). Molecular modeling of clay hydration: A study of hysteresis loops in the swelling curves of sodium montmorillonites. Langmuir.

[12] Chang F.R.C., Skipper N.T., Sposito G. (1995). Computer simulation of interlayer molecular structure in sodium montmorillonite hydrates. Langmuir.

[13] Boek E.S., Coveney P.V., Skipper N.T. (1995). Monte Carlo molecular modeling studies of hydrated Li-, Na-, and K-smectites_Understanding the role of potassium as a clay swelling inhibitor. J. Am. Chem. Soc..

[14] Marry V., Turq P., Cartailler T., Levesque D. (2002). Microscopic simulation of structure and dynamics of water and counterions in a monohydrated montmorillonite. J. Chem. Phys..

[15] Kalinichev A., Liu X., Cygan R. (2016). Introduction to a Special Issue on Molecular Computer Simulations of Clays and Clay–water Interfaces: Recent Progress, Challenges, and Opportunities. Clays Clay Miner..

[16] Seppälä A., Puhakka E., Olin M. (2018). Effect of layer charge on the crystalline swelling of Na+, K+ and Ca^2+^ montmorillonites: DFT and molecular dynamics studies. Clay Miner..

[17] Miranda-Pascual M.G., Chávez-García M.L. (2014). Monte Carlo molecular simulation of the Na-, Mg-, and mixtures of Na/Mg-montmorillonites systems, in function of the pressure. Mol. Phys..

[18] Zhang L., Lu X., Liu X., Zhou J., Zhou H. (2014). Hydration and Mobility of Interlayer Ions of (Nax, Cay)-Montmorillonite: A Molecular Dynamics Study. J. Phys. Chem. C.

[19] Dai W., Shui Z.H., Li K., Duan P. (2010). Molecular Simulation on Modification of Structure and Thermodynamic Properties of Montmorillonite. Appl. Mech. Mater..

[20] Shen W., Li L., Zhou H., Zhou Q., Chen M., Zhu J. (2018). Effects of charge density on the hydration of siloxane surface of montmorillonite: A molecular dynamics simulation study. Appl. Clay Sci..

[21] Marry V., Dubois E., Malikova N., Durand-Vidal S., Longeville S., Breu J. (2011). Water dynamics in hectorite clays: Influence of temperature studied by coupling neutron spin echo and molecular dynamics. Environ. Sci. Technol..

[22] Malikova N., Cadène A., Dubois E., Marry V., Durand-Vidal S., Turq P., Breu J., Longeville S., Zanotti J.M. (2007). Water Diffusion in a Synthetic Hectorite Clay Studied by Quasi-elastic Neutron Scattering. J. Phys. Chem. C.

[23] Morodome S., Kawamura K. (2011). In X-ray Diffraction Study of the Swelling of Montmorillonite as Affected by Exchangeable Cations and Temperature. Clays Clay Miner..

[24] Shapley T.V., Molinari M., Zhu R., Parker S.C. (2013). Atomistic Modeling of the Sorption Free Energy of Dioxins at Clay–Water Interfaces. J. Phys. Chem. C.

[25] Xu J., Camara M., Liu J., Peng L., Zhang R., Ding T. (2017). Molecular dynamics study of the swelling patterns of Na/Cs-, Na/Mg-montmorillonites and hydration of interlayer cations. Mol. Simul..

[26] Sun L., Ling C.Y., Lavikainen L.P., Hirvi J.T., Kasa S., Pakkanen T.A. (2016). Influence of layer charge and charge location on the swelling pressure of dioctahedral smectites. Chem. Phys..

[27] Dazas B., Lanson B., Delville A., Robert J.-L., Komarneni S., Michot L.J., Ferrage E. (2015). Influence of Tetrahedral Layer Charge on the Organization of Interlayer Water and Ions in Synthetic Na-Saturated Smectites. J. Phys. Chem. C.

[28] Christidis G.E., Blum A.E., Eberl D.D. (2006). Influence of layer charge and charge distribution of smectites on the flow behaviour and swelling of bentonites. Appl. Clay Sci..

[29] Sun L., Tanskanen J.T., Hirvi J.T., Kasa S., Schatz T., Pakkanen T.A. (2015). Molecular dynamics study of montmorillonite crystalline swelling: Roles of interlayer cation species and water content. Chem. Phys..

[30] Yi H., Zhang X., Zhao Y., Liu L., Song S. (2016). Molecular dynamics simulations of hydration shell on montmorillonite (001) in water. Surf. Interface Anal..

[31] Liu X., Zhu R., Ma J., Ge F., Xu Y., Liu Y. (2013). Molecular dynamics simulation study of benzene adsorption to montmorillonite: Influence of the hydration status. Colloids Surf. A Physicochem. Eng. Asp..

[32] Qu X., Zhang Y., Li H., Zheng S., Zhu D. (2011). Probing the specific sorption sites on montmorillonite using nitroaromatic compounds and hexafluorobenzene. Environ. Sci. Technol..

[33] Berghout A., Tunega D., Zaoui A. (2010). Density Functional Theory (DFT) Study of the Hydration Steps of Na^+^/Mg^2+^/Ca^2+^/Sr^2+^/Ba^2+^-Exchanged Montmorillonites. Clays Clay Miner..

[34] Cygan R.T., Romanov V.N., Myshakin E.M. (2012). Molecular Simulation of Carbon Dioxide Capture by Montmorillonite Using an Accurate and Flexible Force Field. J. Phys. Chem. C.

[35] Ferrage E., Lanson B., Sakharov B.A., Geoffroy N., Jacquot E., Drits V.A. (2007). Investigation of dioctahedral smectite hydration properties by modeling of X-ray diffraction profiles: Influence of layer charge and charge location. Am. Mineral..

[36] Zhu R., Chen W., Shapley T.V., Molinari M., Ge F., Parker S.C. (2011). Sorptive characteristics of organomontmorillonite toward organic compounds: A combined LFERs and molecular dynamics simulation study. Environ. Sci. Technol..

[37] Zhao Q., Burns S.E. (2012). Molecular dynamics simulation of secondary sorption behavior of montmorillonite modified by single chain quaternary ammonium cations. Environ. Sci. Technol..

[38] Casewit C.J.C.K.S., Rappe A.K. (1992). Application of a Universal Force Field to Main Group Compounds. J. Am. Chem. Soc..

[39] Zhou J., Lu X., Wang Y., Shi J. (2002). Molecular dynamics study on ionic hydration. Fluid Phase Equilibria.

[40] Tambach T.J., Bolhuis P.G., Hensen E.J., Smit B. (2006). Hysteresis in clay swelling induced by hydrogen bonding: Accurate prediction of swelling states. Langmuir.

[41] Tambach T.J., Hensen E.J.M., Smit B. (2004). Molecular Simulations of Swelling Clay Minerals. J. Phys. Chem. B.

[42] Douillard J.M., Lantenois S., Prelot B., Zajac J., Henry M. (2008). Study of the influence of location of substitutions on the surface energy of dioctahedral smectites. J. Colloid Interface Sci..

[43] Mohammadi M., Mohammadiarani H., Shaw V.S., Neubig R.R., Vashisth H. (2019). Interplay of cysteine exposure and global protein dynamics in small-molecule recognition by a regulator of G-protein signaling protein. Proteins.

